# Thalidomide interaction with inflammation in idiopathic pulmonary fibrosis

**DOI:** 10.1007/s10787-023-01193-1

**Published:** 2023-03-25

**Authors:** Nikitha Naomi Dsouza, Varun Alampady, Krishnaprasad Baby, Swastika Maity, Bharath Harohalli Byregowda, Yogendra Nayak

**Affiliations:** Department of Pharmacology, Manipal College of Pharmaceutical Sciences, Manipal Academy of Higher Education, Manipal, Karnataka 576104 India

**Keywords:** Thalidomide, Idiopathic pulmonary fibrosis, Drug repurposing, Cereblon, Anti-inflammatory

## Abstract

The “Thalidomide tragedy” is a landmark in the history of the pharmaceutical industry. Despite limited clinical trials, there is a continuous effort to investigate thalidomide as a drug for cancer and inflammatory diseases such as rheumatoid arthritis, lepromatous leprosy, and COVID-19. This review focuses on the possibilities of targeting inflammation by repurposing thalidomide for the treatment of idiopathic pulmonary fibrosis (IPF). Articles were searched from the Scopus database, sorted, and selected articles were reviewed. The content includes the proven mechanisms of action of thalidomide relevant to IPF. Inflammation, oxidative stress, and epigenetic mechanisms are major pathogenic factors in IPF. Transforming growth factor-β (TGF-β) is the major biomarker of IPF. Thalidomide is an effective anti-inflammatory drug in inhibiting TGF-β, interleukins (IL-6 and IL-1β), and tumour necrosis factor-α (TNF-α). Thalidomide binds cereblon, a process that is involved in the proposed mechanism in specific cancers such as breast cancer, colon cancer, multiple myeloma, and lung cancer. Cereblon is involved in activating AMP-activated protein kinase (AMPK)-TGF-β/Smad signalling, thereby attenuating fibrosis. The past few years have witnessed an improvement in the identification of biomarkers and diagnostic technologies in respiratory diseases, partly because of the COVID-19 pandemic. Hence, investment in clinical trials with a systematic plan can help repurpose thalidomide for pulmonary fibrosis.

**Graphical Abstract:**

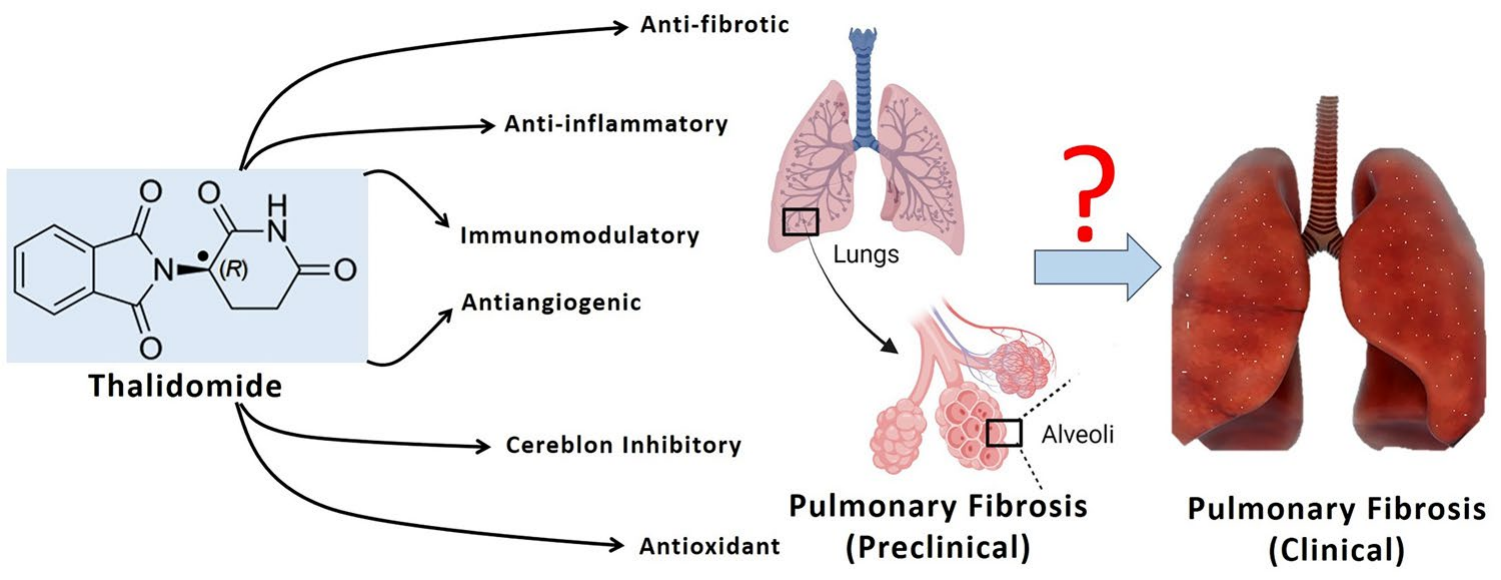

## Introduction

Idiopathic pulmonary fibrosis (IPF) is a progressive interstitial lung disease characterized by chronic fibrotic scarring of lung tissues, leading to a deterioration of lung function and respiratory failure. Disease progression ultimately leads to death within a few years. IPF is presumably caused by multiple causes such as smoking, drugs (E.g., bleomycin), inhalation of metallic particles, wood dust or silica, and exaggerated immune responses (Barratt et al. 2018). Environmental factors and epigenetic mechanisms such as DNA-methylation and histone modifications are major contributors to disease progression (Velagacherla et al. 2022). IPF is an abnormal wound healing process where micro-injuries to the alveolar epithelium do not heal appropriately (Richeldi et al. 2017). IPF is a chronic fibrotic disease and symptomatically overlaps with cryptogenic fibrosing alveolitis. In IPF, an altered extracellular matrix (ECM) replaces the healthy lung tissue, damaging alveolar integrity, which is reflected by decreased lung compliance, abnormal gas exchange, respiratory failure, and eventually, death. IPF histopathology reflects interstitial pneumonia with fibroblast foci as hallmark lesions (Martinez et al. 2017). IPF is caused by sustained micro-injuries to the alveolar epithelial tissue, combined with abnormal healing processes leading to the depletion of the basement membrane between the alveolar epithelium and the capillaries (Wuyts et al. 2020). IPF is most prevalent in South Korea, Canada, Poland, the United States, and Italy (Maher et al. 2021). The COVID-19 pandemic could have augmented the growing incidences of IPF (Tanni et al. 2021). The pathogenesis of post-COVID-19 pulmonary fibrosis involves TGF-β1, which facilitates the release of extracellular proteins, fibroblast proliferation, fibroblast migration and, myofibroblast conversion (Al-kuraishy et al. 2022). Furthermore, genetic susceptibility for IPF and COVID-19 seem to be similar, as confirmed by the fibrotic plasma biomarkers such as thrombospondin 2 (TSP2), glial-derived factor 15 (GDF15), insulin-like growth factor binding protein 7 (IGFBP7), and procollagen type III (PROC3) (Ackermann et al. 2022). Currently, the therapeutic management of IPF consists of symptomatic treatment and extensive palliative care, because nintedanib and pirfenidone are the only two drugs approved for the indication of IPF (Mudawi et al. 2021). In this review, we propose that thalidomide can mitigate inflammation in IPF, and can be repurposed either as monotherapy or in combination with nintedanib and pirfenidone.

**Fig. 1:**
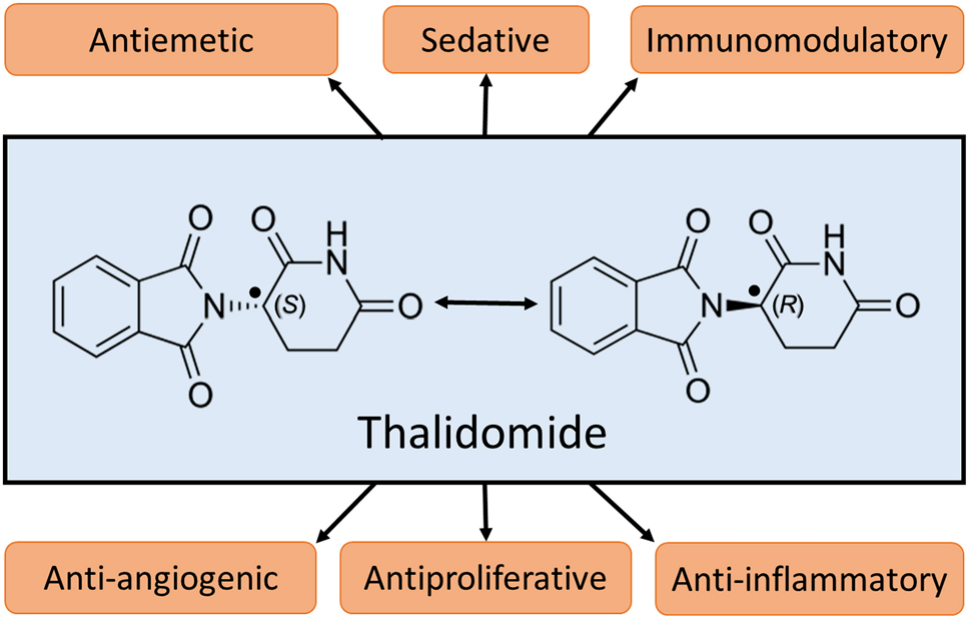
Structure of thalidomide showing its R( +) and S(-) enantiomers and its actions. The structure of thalidomide has asymmetric carbon (•); Both R( +) and S( −) enantiomers are equally active as they are interconvertible in the body

Thalidomide is a synthetic glutamic acid derivative α-(N-phthalimido)-glutarimide (Fig. 1), originally designed as a non-addictive and non-barbiturate sedative. (Kim and Scialli 2011). Thalidomide has one asymmetric carbon in the glutarimide ring, which acts as a chiral centre and gives rise to two enantiomers, R(+) and S(−). These enantiomers are capable of interconverting under appropriate biological conditions (Vargesson 2015). Being hailed as a ‘wonder drug’ at the time of its introduction, thalidomide was used as a quick and effective anti-emetic to treat morning sickness in pregnant women (Sharma and Kwatra 2016). It appeared to be non-toxic in rodent models. Hence, an LD_50_ value was not established, and it was assumed to be safe for humans (Cooper et al. 2010). Thalidomide received a lot of attention for being inexpensive and easily available without prescription. Thalidomide was widely marketed in European countries, Australia and Japan. It was first marketed in West Germany in 1957 under the trade name Contergan VR by Chemie Grunenthal, a German pharmaceutical company, and was later approved and distributed in 46 countries around the world under various brand names (Kumar et al. 2012). In 1960, around 14.6 tonnes of thalidomide were believed to be sold in Germany alone, making it the best-selling sedative in the market (Rehman et al. 2011). However, what came next was one of the most catastrophic man-made clinical disasters in history. There were multiple cases of limb malformations, birth defects and even death of thousands of babies born to mothers who consumed thalidomide during their early trimester of pregnancy (Kim and Scialli 2011). Thalidomide was withdrawn from the market in September 1961 when two scientists, McBride in Australia and Lenz in Germany, confirmed the association of thalidomide with congenital disabilities such as limb deformities, congenital cardiac, renal and other organ malformations (Mellin and Katzenstein 1962). Although thalidomide was swiftly withdrawn from the market, more than 10,000 infants suffered from congenital defects, with the survivors still suffering even today (Newbronner et al. 2019). However, the drug thalidomide is still an interesting molecule in drug discovery because of its anti-inflammatory and immunomodulatory properties and is now classified along with immunomodulatory imide drugs (IMiDs) such as lenalidomide, pomalidomide, apremilast, 3,6′-dithiopomalidomide, 3,6′-dithiothalidomide, N-adamantyl phthalimidine, and dithiophthalimide (Furihata et al. 2020). Some of these IMiDs are proposed for repurposing in neuropsychiatric and neurodegenerative disorders (Jung et al. 2021).

### Therapeutic actions which enabled the re-entry of thalidomide

Due to the teratogenic effects, thalidomide was withdrawn late in November 1961 (Samel et al. 2019). However, the journey of thalidomide did not end there. In 1965, when thalidomide was administered as a sedative to patients with leprosy (Sheskin 1965), it was observed that along with its sedative action, thalidomide also reduced elevated body temperature, and stimulated the reabsorption of erythema nodosum-like skin lesions in patients with leprosy (Sheskin 1965). Thus, it was reintroduced into the market with extreme caution for the treatment of erythema nodosum leprosum, based on its immunomodulatory, anti-angiogenic, and anti-inflammatory actions. In the 1980s, thalidomide was given to patients with classic and definitive rheumatoid arthritis (Gutiérrez-Rodríguez 1984). In these patients, the erythrocyte sedimentation rate reportedly returned to normal within a few weeks, pain and joint inflammation disappeared, and the rheumatoid factor titers had significantly decreased (Gutierrez-Rodriguez et al. 1989). In 1986, a clinical study was conducted on the effect of thalidomide in 30 male patients suffering from Behçet’s disease, which is characterized by inflammation of the blood vessels in the body (Hamza 1986). The results demonstrated that thalidomide could be useful in treating clinical manifestations of Behçet’s disease. The side effects observed included skin rashes and somnolence, and were uncommon (Hamza 1986).

Thalidomide also selectively inhibited tumour necrosis factor-alpha (TNF-α) production in lipopolysaccharide-stimulated monocytes (Piura et al. 2013). However, there was no change in the levels of granulocytes / macrophage colony-stimulating factor, and cytokines [interleukin-6 (IL-6) and interleukin-1-beta (IL-1β)] produced by the monocytes. This could be due to increased degradation of TNF-α mRNA. This allowed the use of thalidomide in the treatment of severe inflammatory diseases (Majumder et al. 2012). Thalidomide has also been used in the treatment of wasting syndrome in HIV-AIDS patients (Reyes-Terán et al. 1996). In addition, the anti-TNF-α action of thalidomide was considerably helpful in treating tuberculous meningitis in children (van Toorn et al. 2021). An in vivo experiment in rabbits concluded that thalidomide is a potent inhibitor of angiogenesis (D’Amato et al. 1994). Among the putative angiogenesis inhibitors tested, thalidomide was the only drug that was effective orally suggesting that thalidomide is a feasible anticancer agent (Zhu et al. 2021). Thalidomide has also shown promising activity against various solid tumours such as breast cancer, renal cancer, Kaposi’s sarcoma, liver cancer, lung cancer, and several glioblastomas (Amare et al. 2021). US FDA authorized thalidomide for the treatment of erythema nodosum leprosum in 1998, and multiple myeloma in 2006 (Rehman et al. 2011). Its use in multiple myeloma was attributed to the immunomodulatory and anti-angiogenic activity which downregulates the expression of TNF-α and IL-6 by the stromal cells in the bone marrow, thereby inhibiting the proliferation of multiple myeloma cells (Holstein and McCarthy 2017). A recent compilation by Barbarossa et al. listed more than 130 analogues of thalidomide that were tested to treat cancer (Barbarossa et al. 2022). Efforts have now been made to strictly regulate the use of thalidomide to prevent potential harm from thalidomide. Several programmes have been developed to manage this. Risk Evaluation and Mitigation Strategies is a programme that controls the prescription of thalidomide (Brandenburg et al. 2017). The System for Thalidomide Education and Prescribing Safety (STEPS) is yet another initiative designed to supervise the distribution and use of thalidomide (Khalil et al. 2020).

#### Unfolding the mechanism of action of thalidomide for drug repurposing

Understanding the mechanism of teratogenicity is crucial for safer repurposing of thalidomide and its analogues for therapeutic applications. Soon after the horrifying thalidomide tragedy, research began on the teratogenicity of the drug. In 1979 (Blaschke et al. 1979), after thorough research on the thalidomide enantiomers, it was established that the (R)- and (S)- enantiomers possess varying biological characteristics, with the teratogenicity induced only by the (S)-enantiomer. No embryopathy was reported when the (R)- enantiomer was tested in animals (Tokunaga et al. 2018). There were several hypotheses proposed to explain the abnormal foetal development caused by thalidomide. One such theory was that thalidomide induces oxidative stress, thereby altering the NF-ĸB activity, causing impaired limb formation and other complications in embryos (Hansen et al. 2002). Another theory attributed the teratogenicity to the anti-angiogenic action of thalidomide, suggesting that there is a downregulation of growth factors and aberrant blood vessel formation at the limbs (D’Amato et al. 1994). However, these theories failed to elaborate on the targets of thalidomide and how these targets regulate its action (Asatsuma-Okumura et al. 2020).

**Fig. 2:**
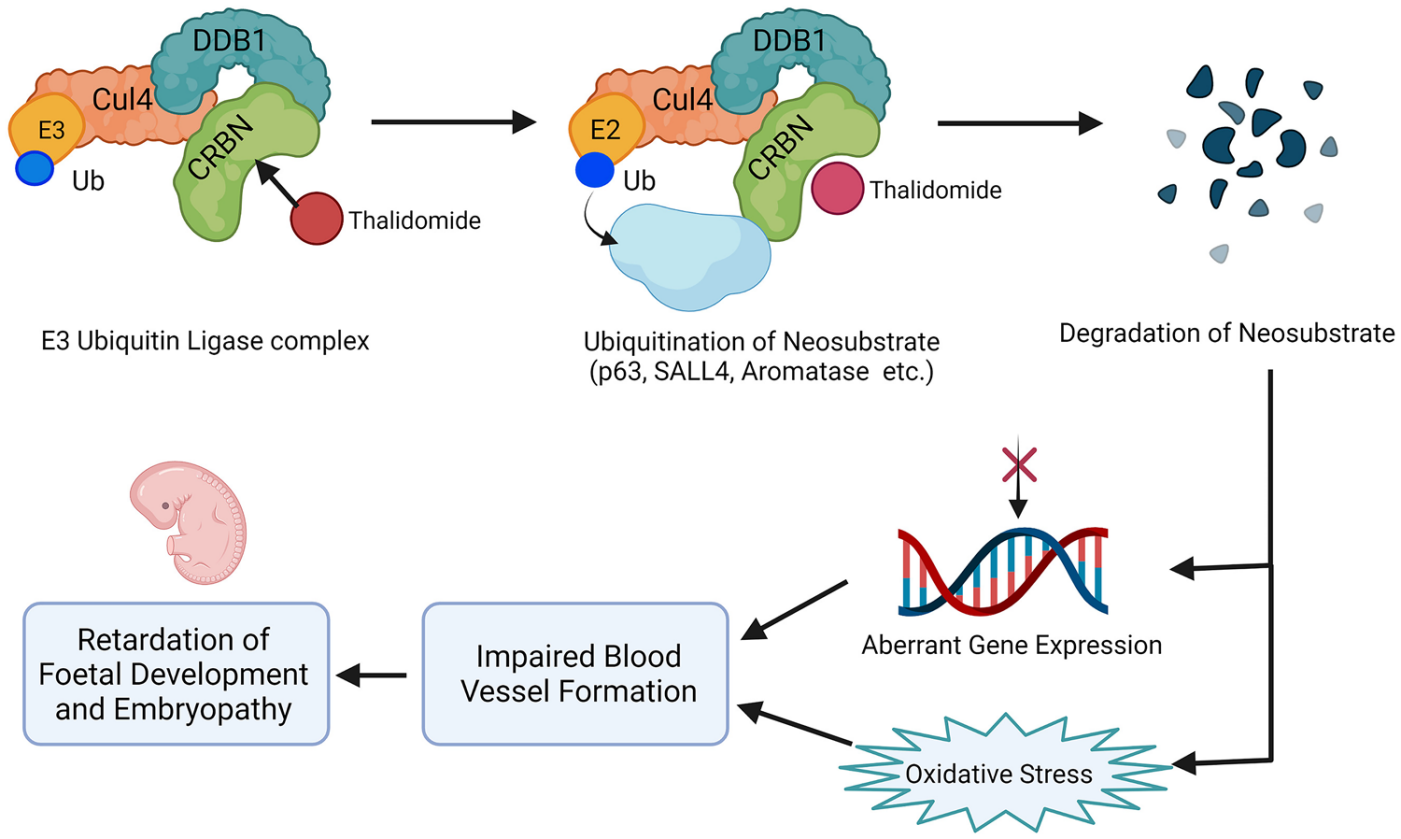
Mechanism of thalidomide-induced teratogenicity. Thalidomide binds to cereblon (CRBN), which is a part of the E3-ubiquitin ligase complex with the damaged DNA binding protein 1 (DDB1), and cullin-4 (Cul4) forming Cullin-RING Ligase-4 (CRL4CRBN) complex. This leads to an interruption in the normal functioning of cereblon and recruitment of ‘neosubstrates’ such as SALL4 and p63 for degradation, initiating altered gene expression and subsequent limb malformations. Figures are created with created with BioRender.com

In 2010, a breakthrough study identified that thalidomide binds to a protein called cereblon, linked to a crucial gene for mental retardation (Ito et al. 2010). Cereblon was recognized as the primary target for thalidomide. Cereblon is known to bind to another protein called damaged DNA binding protein 1 (DDB1) (Ito et al. 2011). Further, the DDB1 and Cul4 (cullin-4) configure an E3-ubiquitin ligase complex called Cullin-RING Ligase-4 (CRL4^CRBN^) (Chang and Keith Stewart 2011). This complex plays a vital role in the limb formation and expression of growth factors necessary for limb development (Ito and Handa 2012). Thus, it was concluded that the primary targets of thalidomide are (a) cereblon, which interacts directly with thalidomide, and (b) DDB1, which then interacts indirectly by its binding to cereblon (Ito et al. 2010). By binding to cereblon, thalidomide interferes with the normal functioning of the ligase complex and causes the degradation of certain neosubstrates, thus altering the expression of genes related to embryonic growth, leading to foetal abnormalities (Fig. 2) (Gao et al. 2020). Therefore, thalidomide and its analogues are now considered as cereblon-modulating drugs, especially for cancer treatment (Asatsuma-Okumura et al. 2019b). Further, Ito and Handa (2020) have explained the old and the current model of thalidomide mechanism of action to induce teratogenicity and its exploration in recent drug development (Ito and Handa 2020). Thalidomide downregulates fibroblast growth factor 8 (FGF8), thereby upregulating the apoptotic genes in the limbs (Kawamura et al. 2014). Therefore, the FGF8 downregulation could be another molecular mechanism that explains the abnormally short limbs in deformed infants exposed to thalidomide during embryonic development (Asatsuma-Okumura et al. 2019a). The cereblon protein is found primarily in the brain, particularly the cerebellum. Mutations in the cereblon gene is associated with mental retardation, compromising cognitive processes and memory (Choi et al. 2018). Apart from the brain, cereblon is largely present in the kidney, lung, liver, intestines, placenta, and prostate. Further, cereblon engages in the regulation of several other processes, such as the regulation of calcium and chloride channels (Kim et al. 2016). Cereblon also acts as a substrate receptor protein in the E3-ubiquitin ligase complex and determines the specificity of the substrate-binding to the complex. Hence, cereblon is pivotal in handling the degradation of many substrates (Liu et al. 2015). However, the recent study by Kowalski et al., also says that the cereblon mechanism vary highly between 14 species. Thalidomide does not have teratogenic effect in mice, while limb anomalies are absent in rats. In rabbits and sheep, thalidomide produces teratogenic effects, and the teratogenic characteristics in Rhesus monkeys are similar to that of humans (Kowalski et al. 2021).

#### Etiopathogenesis and role of cereblon in pulmonary fibrosis

**Fig. 3:**
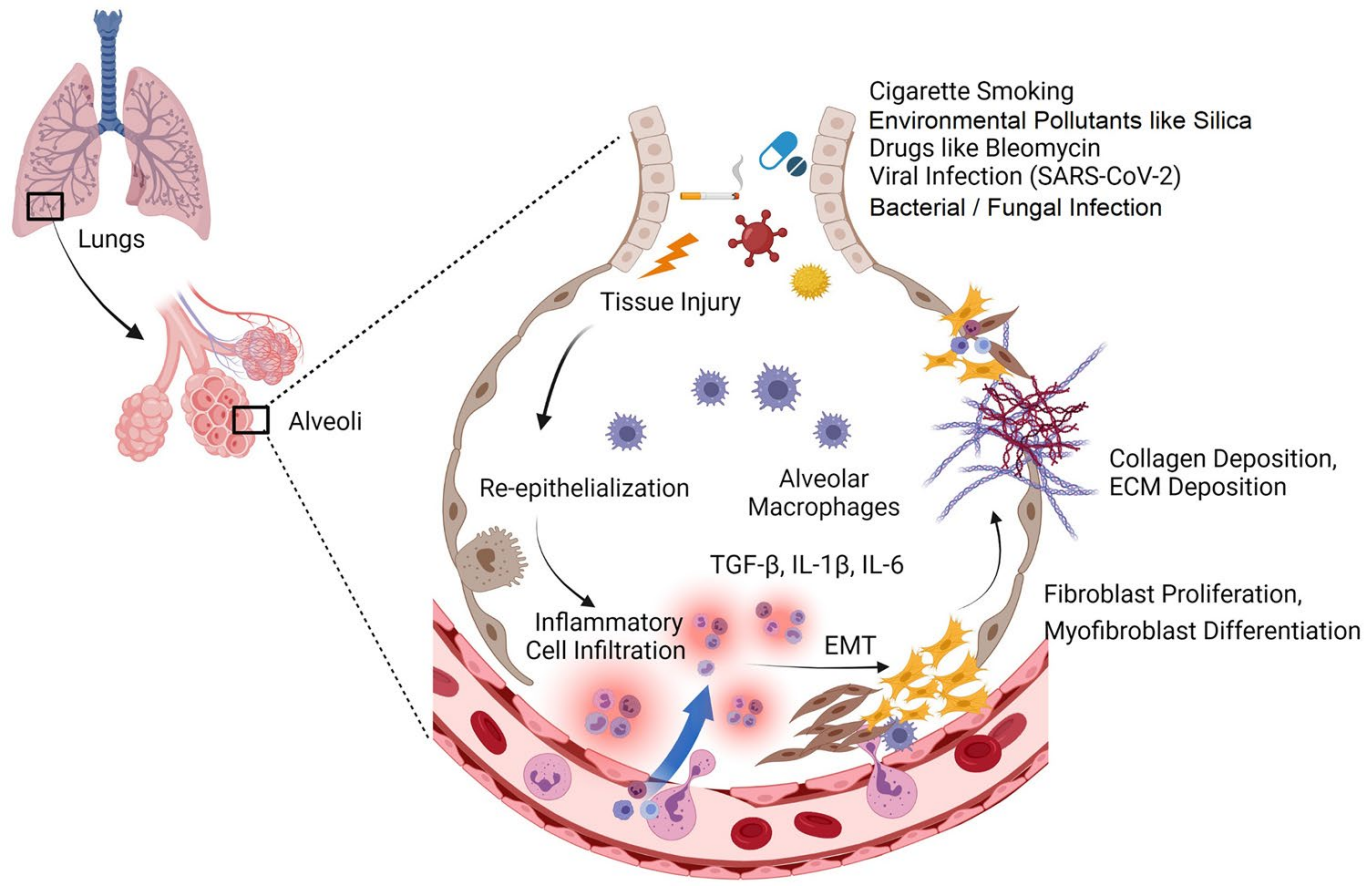
Etiopathogenesis of pulmonary fibrosis. Repeated tissue injuries cause AECs to undergo apoptosis and re-epithelialization, causing the recruitment of inflammatory cells and profibrogenic mediators, and subsequent ECM accumulation, leading to fibrosis. ECM: Extracellular matrix, EMT: Epithelial mesenchymal transition. Figures are created with created with BioRender.com

Despite extensive research over the past years, the pathogenesis of IPF remains unclear. Recent advances have linked the disease to inflammation and oxidative stress, which occurs in response to tissue injury. Generally, several endogenous and external factors contribute to tissue injury and damage the alveolar epithelium. Genetic factors such as polymorphisms of the surfactant protein or variations in the mucin 5B gene (*MUC5B/Muc5b*) (Michalski and Schwartz 2020), environmental factors such as inhalation of irritant dust particles, microbial infections, lifestyle habits such as cigarette smoking, are some of the attributable factors (Kärkkäinen et al. 2017; Denver et al. 2018). The aetiology of the most common lung fibrotic condition, IPF, is still not clearly understood. However, the genetic and epigenetic factors equally contribute to the etiopathogenesis of IPF (Velagacherla et al. 2022). The telomerase reverse transcriptase (*TERT*) gene is involved in more than 50% of IPF patients (van Batenburg et al. 2020). *TERT* is highly expressed in chronic smokers, which signifies the role of epigenetics (Diaz de Leon et al. 2010). What was earlier thought to be chronic inflammation is now being attributed to an aberrant wound healing in genetically susceptible individuals (Daccord and Maher 2016). It is now believed that there is dysregulation at any of the three processes: (i) injury (ii) inflammation (iii) repair, along with the overexpressed pro-fibrotic mediators such as TGF-β, and IL-1β. This leads to cellular and molecular modifications that develop fibrosis by the formation of myofibroblasts and deposition of ECM components such as collagen in the tissues (Wilson and Wynn 2009; Cinausero et al. 2017). Research has established that epigenetic factors and sustained injuries to the alveolar epithelial layer lead to the activation of alveolar epithelial cells (AECs). These activated AECs further engage in the attraction of inflammatory cells and promote fibrogenesis by activating fibroblasts. The fibroblasts differentiate into myofibroblasts and facilitate the process of fibrosis (Magnini et al. 2017) (Fig. 3).

Recent research on the implications of cereblon in IPF has opened up a new drug target (Kang et al. 2021). Cereblon modulates the energy metabolism by negatively regulating the activation of the AMP-activated protein kinase (AMPK)-signalling pathway (Lee et al. 2011). Thus, the role of cereblon-AMPK signalling pathway in IPF can be explored for drug discovery. In recent studies, AMPK activators such as metformin, have shown a protective effect against IPF by preventing the TGF-β/Smad signalling pathway and release of collagen, fibronectin, α-smooth muscle actin (α-SMA), and other fibrogenic factors (Rangarajan et al. 2018; Cheng et al. 2021; Wu et al. 2022). Therefore, activation of AMPK may inhibit the TGF-β-induced fibroblast proliferation (Chen et al. 2022). AMPK has also shown an inhibitory action on Forkhead box protein M1 (FOXM1), which is a crucial transcription factor in the process of cell proliferation. FOXM1 is now found to play a role in activating fibroblasts and their proliferation (Gu et al. 2021). The activation of AMPK also decreased the endoplasmic reticulum stress and unfolded protein response, both of which are driving factors for the development of fibrosis by inducing tissue injury in the alveolar epithelium (Choi et al. 2016; Burman et al. 2018). A computational investigation of a well-known AMPK activator DHFO, having structural features that are slightly different from thalidomide was reported to have better CRBN binding affinity than thalidomide (Nayek et al. 2022), Thus, the activation of the AMPK signalling pathway is crucial for protecting against IPF.

**Fig. 4:**
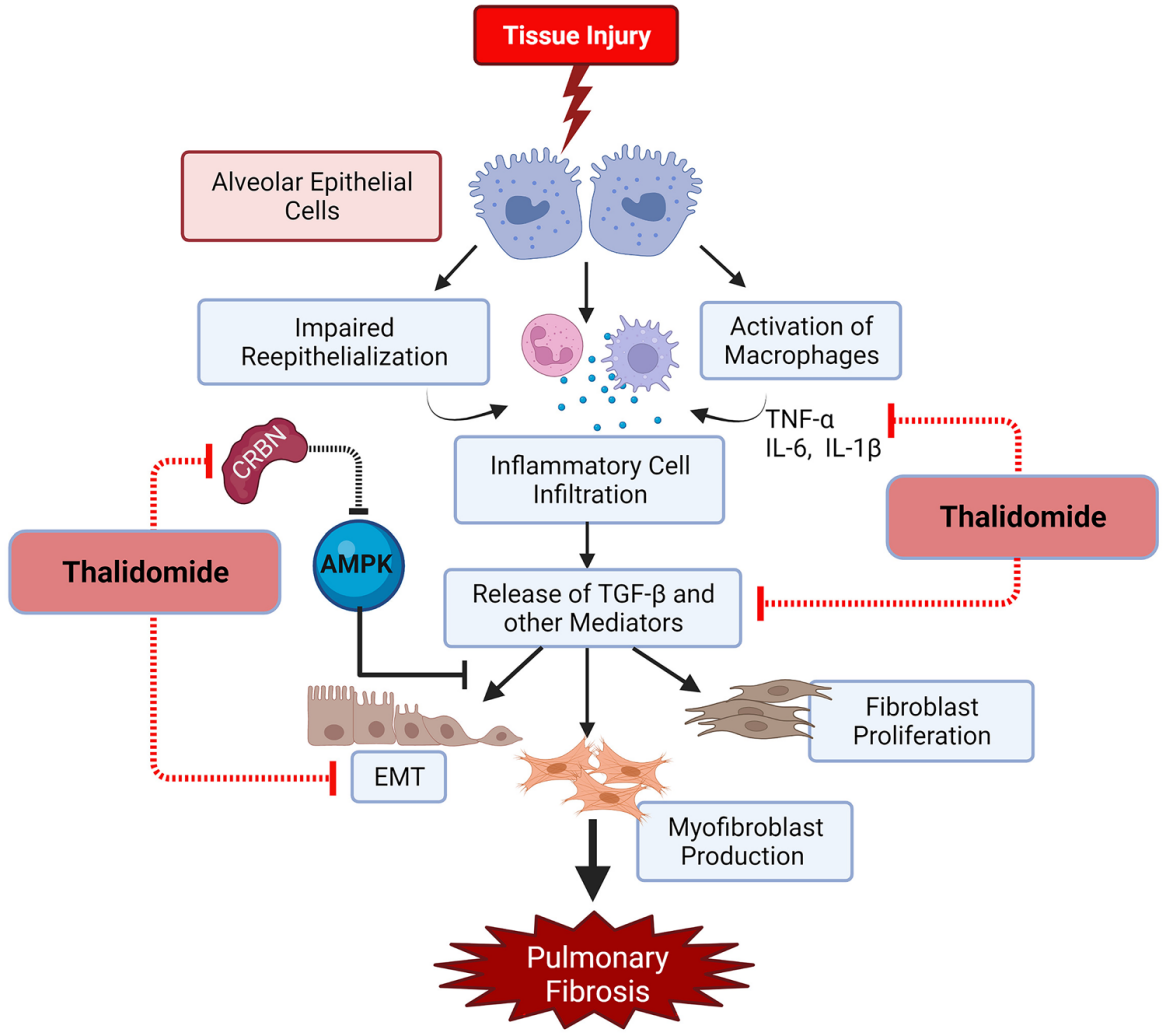
Role of cereblon and actions of thalidomide in pulmonary fibrosis. Overexpression of cereblon (CRBN) leads to the downregulation of AMPK, which otherwise inhibits TGF-β-induced fibroblast proliferation, myofibroblast differentiation and epithelial-mesenchymal transition (EMT). Thus, CRBN inhibits the protective actions of AMPK against pulmonary fibrosis. By inhibiting *CRBN* thalidomide stops the progression of fibrosis. Figures are created with created with BioRender.com

The overexpression of cereblon would lead to the inactivation of AMPK, and subsequent progression of IPF. Cereblon binds to the α-subunit of AMPK and reduces its overall catalytic activity, which suppresses the activation of AMPK, and worsens IPF (Kang et al. 2021). A recent study conducted by Kang et al. in 2021 has provided a strong basis for the association of cereblon in IPF by showing that the collagen and fibronectin were decreased in cereblon gene (CRBN) knockout mice. On the other hand, the TGF-β-induced phosphorylation and activation of Smad proteins, which occurs through *CRBN* activation, was decreased in the presence of an AMPK activator such as metformin (Kang et al. 2021). Moreover, higher expression of cereblon is also related to increased lung injury by oxidative stress, followed by inflammation. This can be subdued by repressing the expression of cereblon, which will downregulate the activation of the NF-ĸB pathway and its downstream mediators in the lungs (Yang et al. 2020). Hence, modulation or inhibition of cereblon by thalidomide and its analogues could be a novel approach to treating IPF (as depicted in Fig. 4).

#### Current treatment options for pulmonary fibrosis

Currently, there are two approved drugs for IPF available in the clinics, namely pirfenidone and nintedanib. They are well tolerated upon long-term treatment without any newer safety alerts (Cameli et al. 2020). Due to the poor understanding about pathogenesis of IPF, there have been several therapeutic failures over the past decades. This has left us with minimal treatment options for IPF. The earliest approach was to target the resulting inflammatory response using immunomodulators and anti-inflammatory drugs. Drugs targeting inflammatory and immune responses were recommended in the latest update of clinical practice guidelines on the treatment of IPF (Luppi et al. 2021). There are clinical trials conducted on drugs for the treatment of IPF that were later declared potentially harmful (Lota and Wells 2013). One such trial was based on the importance of the coagulation cascade in IPF (Kubo et al. 2005). The drug warfarin did not show any benefit to patients with progressive IPF, and moreover, warfarin treatment was associated with increased mortality in patients with IPF (Noth et al. 2012). Another drug named everolimus, a macrocyclic proliferation signal inhibitor with anti-fibroproliferative activity, was tested for its efficacy in IPF (Malouf et al. 2011). This trial involving 89 patients with IPF, (confirmed by surgical lung biopsy) concluded that everolimus was associated with rapid disease progression (Malouf et al. 2011). One of the widely used treatments for IPF was a combination of prednisone, azathioprine, and N-acetylcysteine. However, the safety of this drug combination was not established. After a 60 week treatment period and 32 weeks follow-up, this three-drug combination was conclusively implicated with an increased risk of hospitalization and death (Raghu et al. 2012). There are quite a few clinical trials of ineffective drugs which made a landmark contribution to IPF patient care. One such clinical trial was on sildenafil, a phosphodiesterase-5 inhibitor. The study on 180 patients treated with sildenafil did not meet its pre-defined significance (Zisman et al. 2010). However, some secondary outcomes, such as the degree of dyspnoea and quality of life, showed minor differences in favour of sildenafil. Another study on a combination of sildenafil and pirfenidone did not show any beneficial effect in preventing pulmonary hypertension associated with IPF (Behr et al. 2021). A prospective, randomized, double-blind, placebo-controlled, parallel-group, event-driven, morbidity–mortality clinical trial of bosentan, (an endothelin receptor antagonist) was conducted on 616 patients to investigate the benefit of modulating pulmonary hypertension associated with IPF (Giordano et al. 2010). However, bosentan failed to show any efficacy because there were no differences between treatment groups in health-related quality of life or dyspnoea (King et al. 2011).

Some of the investigational drugs under clinical development are TD139, PLN-74809, PLN-74809, TRK-250, PRM-151, and pamrevlumab (Glass et al. 2022). The investigational drug TD139 is a small molecule inhibitor of galectin-3 (Hirani et al. 2021). Integrins are known to activate TGF-β and have a role in pulmonary fibrosis (Sheppard 2008). Developmental endothelial locus-1 (Del-1) is an endogenous inhibitor of TGF-β activation (Kim et al. 2020). The newer molecule PLN-74809, a dual selective inhibitor of integrins αVβ1/αVβ6, is an emerging drug of the future (Decaris et al. 2021). PLN-74809 inhibits the deposition of collagen in lung tissue by blocking TGF-β pathway and inhibiting phosphorylation of Smad-3. It has shown efficacy in clinical trials (Decaris et al. 2021). TRK-250, produces siRNA in the body, and targets TGF-β mRNA is in clinical trial phase-1 (NCT03727802). PRM-151, a recombinant human serum amyloid P/pentraxin 2 (PTX2) (Raghu et al. 2019), is currently in phase-3 clinical trials (NCT04552899). The monoclonal antibody pamrevlumab, which targets connective tissue growth factor (CTGF) is currently in phase-2 clinical trial (Richeldi et al. 2020).

#### Drug targets for thalidomide in pulmonary fibrosis

##### Anti-fibrotic action

The potential use of thalidomide in interstitial pulmonary diseases has been debated since early 2000s (Ye 2006). The immunomodulatory action of thalidomide was seen as a desirable attribute with a potential to treat and manage debilitating diseases such as IPF. In addition, over recent years, the antiangiogenic action of thalidomide has also gained the attention of researchers. Thalidomide is considered helpful in fibrotic diseases mainly due to its immunomodulatory, antiangiogenic, antioxidant and anti-inflammatory actions (Ye 2006). Thalidomide is being proven as antifibrotic in bleomycin-induced mouse model of pulmonary fibrosis (Tabata et al. 2007). Inhibition of TGF-β is the major mechanism reported. Furthermore, thalidomide downregulates α-SMA in human foetal lung fibroblasts (Choe et al. 2010). In bleomycin-induced pulmonary fibrosis, thalidomide downregulates p-JNK and α-SMA (Liu et al. 2014). Dong and colleagues (2017) demonstrated that thalidomide inhibits pulmonary fibrosis both in vitro and in vivo. The same group has also demonstrated that the activity is due to regulation of the enzyme thioredoxin reductase (Dong et al. 2017a). A cell line-based study conducted on Human Embryonic Lung Fibroblasts (HELF) has established that thalidomide is capable of directly exerting an antifibrotic action by inhibiting the activity of connective tissue growth factor (CTGF). CTGF is responsible for cell transformation, angiogenesis, myofibroblast differentiation and even upregulated ECM synthesis in the fibroblasts (Wu et al. 2020). This study proved that it was safer to block CTGF using thalidomide, than to block TGF-β altogether to prevent the pro-fibrotic actions of TGF-β. Thalidomide has also shown antifibrotic activity in the kidneys of streptozotocin-induced diabetic rats via upregulating the phosphorylation of AMPKα and inhibiting the NF-ĸB and TGF-β1/Smad signalling pathways of inflammation (Zhang et al. 2018). Moreover, it has also prevented the epithelial-mesenchymal transformation (EMT) through TGF-β-induced Smad-independent pathways by suppressing the phosphorylation and activation of several downstream proteins such as ERK, p38, Akt, which ultimately reduce the expression of fibrotic factors such as collagen and α-SMA (Zhou et al. 2017). The process of EMT is also triggered by thalidomide, mediated by EGFL6/PAX6 axis (Tang et al. 2020). Thalidomide has proven to be helpful in diminishing the expression of fibronectin by preventing keloid formation, thus being beneficial in treating skin fibrosis (Liang et al. 2013). With this emerging body of evidence, thalidomide shows potential for use in preventing fibrotic progression in pulmonary fibrosis.

#### Immunomodulatory and anti-inflammatory actions

The pathological evolution of IPF is strongly regulated by the entry of inflammatory cytokines and inflammatory mediators, such as IL-6, IL-1β, TNF-α, and TGF-β, into the alveolar space (Akdis et al. 2016). Thalidomide exerts anti-inflammatory effect by selectively inhibiting the production of TNF-α and other cytokines such as IL-12p40, IL-18, and IL-8 produced by the alveoli macrophages upon stimulation (Sampaio et al. 1991; Ye 2006). Thalidomide shows immunomodulatory action by preventing the activation of immune cells such as Th1 cells and related cytokines (Horton and Hallowell 2012). Choe et al*.* reported that thalidomide successfully inhibits the ERK1/2 pathways of signalling, which are induced by TGF-β in bleomycin-induced pulmonary fibrosis in mice (Choe et al. 2010). TGF-β is an important marker of tissue fibrosis that regulates the production of ECM components such as fibronectin and collagen (Kim et al. 2018). Thalidomide inhibits the pathways downstream of TGF-β, such as p38/Smad signalling, ERK 1/2 signalling, and MAPK pathways, by preventing the phosphorylation and subsequent activation of the proteins involved in the pathways (Liang et al. 2013; Zhou et al. 2017). The recent findings on IMiDs such as lenalidomide and pomalidomide promote the recruitment of aromatase to cereblon, resulting in the degradation of aromatase by proteasomes in immune cells (Tochigi et al. 2020). El-Zahabi et al. 2020, have synthesized more than 40 thalidomide analogues, which were immunomodulators and ameliorated human TNF-α, caspase-8 (CASP8), human vascular endothelial growth factor (VEGF) and nuclear factor-kappa-BP65 (NF-κBP65) in human colon cancer (HCT-116) cells. Further, these derivatives have been shown to be immunomodulatory anticancer agents (El-Zahabi et al. 2020).

#### Antiangiogenic action

Since the anti-angiogenic action of thalidomide was established in 1994, thalidomide has been considered a potential drug candidate for treating several types of cancers and other metabolic disorders that require the inhibition of blood supply to tumours, or correct a pathologic angiogenic state (Behl et al. 2017). In addition, it is possible to extend this theory to the treatment of IPF. The role of angiogenesis in IPF has been constantly debated. It is still unclear how the aberrant neovascularization in the alveolar epithelium is related to the progression of IPF. However, nintedanib, a tyrosine kinase inhibitor, shows its antifibrotic action by targeting vascular endothelial growth factor receptors (VEGFR), platelet-derived growth factor (PDGF), and fibroblast growth factor (FGF)-receptors, all of which are involved in angiogenic signalling pathways (Ackermann et al. 2017). This indicates the possibility of using thalidomide as an anti-angiogenic agent in the treatment of IPF. A study conducted by Tabata et al*.* reported successful prevention of bleomycin-induced pulmonary fibrosis in mice by the anti-angiogenic action of thalidomide. It was observed that thalidomide, in addition to inhibiting angiogenesis in mice lung tissue, also reduced the expression of IL-6, TGF-β_1_, VEGF, Ang-1 Ang-2, and COL1A1 mRNA (Tabata et al. 2007). Thalidomide targets epidermal growth factor-like domain multiple 6 (EGFL6) and inhibits angiogenesis through EGFL6/PAX6 axis (Tang et al. 2020). The effect of thalidomide on EGFL6 is mediated through cereblon protein interaction with protease Lon N-terminal peptide, and this process is mediated by proteasome (Tang et al. 2020). Thalidomide and other IMiDs have reportedly reduced cell migration and adhesion by inhibiting the VEGF receptor, thereby reducing the capillary density on fibrotic lesions (Domingo et al. 2020), demonstrating that thalidomide could possibly be used as an anti-angiogenic for managing IPF.

#### Cereblon inhibitory action

*CRBN* has been established as the main target of thalidomide and its analogues (Ito et al. 2010). On binding to *CRBN*, thalidomide inhibits the activity of the E3-ubiquitin ligase of the multiple-protein complex CRBN-DDB1-Cul4 (Shortt et al. 2013). Cereblon is also involved in the pathogenesis of IPF (Kang et al. 2021). Thalidomide and other IMiDs such as lenalidomide and pomalidomide inhibit the action of cereblon and allow the expression of AMPK (Yang et al. 2021). Inhibition of AMPK is one of the mechanisms by which metformin inhibits proliferation and differentiation of human foetal lung fibroblast (Gu et al. 2021; Chen et al. 2022). AMPK modulation through cereblon shows a protective action and slows down the growth of fibrogenic proteins such as fibronectin, collagen α-SMA. Further, AMPK is down-regulated by the CRL4A-CRBN axis through the polyubiquitination of AMPKα isoforms (Kwon et al. 2019). Thus, cereblon inhibition could be the significant mechanism in mitigating fibrosis.

##### Antioxidant action

There is compelling evidence to implicate oxidative stress as one of the molecular mechanisms in IPF. Oxidative stress occurs from the imbalance between generation of reactive oxygen species (ROS) and the body’s ability to detoxify them (Todd et al. 2012; Estornut et al. 2022). Antioxidant enzymes (such as superoxide dismutase (SOD), catalase, glutathione peroxidase), and malondialdehyde (MDA), in the tissue homogenate are the markers of oxidative stress (Tsikas 2017). MDA accounts for lipid peroxidation or the extent of tissue damage occurring at the alveolar basement membrane (Tsikas 2017). The current standard drug pirfenidone demonstrated to have good antioxidant defence in human pulmonary artery smooth muscle cells (HPASMCs) (Fois et al. 2018). Further, pirfenidone and nintedanib exert beneficial effects on oxidative stress markers in humans (Fois et al. 2020; Li et al. 2022a; Wang and Qu 2022). In a study conducted by Dong et al. in 2017, treatment with thalidomide significantly increased SOD and normalized MDA and ROS in bleomycin-induced mouse model of pulmonary fibrosis. In another study, thalidomide significantly reduced MDA and ROS to near normal, and SOD was increased (Dong et al. 2017a). In IPF, the antioxidant defences are regulated significantly through the redox-sensitive transcription factor nuclear factor, erythroid-derived 2 (Nrf2) (Wang et al. 2022; Dong et al. 2022). The Nrf2 binds to specific antioxidant response elements (AREs), stimulates the antioxidant enzyme genes, and regulates their expression (Walters et al. 2008; Liu et al. 2019). An *in vivo* study conducted in a bleomycin-induced mice model demonstrated the abilities of pirfenidone to balance the antioxidant defence or regulate the oxidative stress through Nrf2/Bach1 equilibrium (Liu et al. 2017). Similarly, thalidomide attenuated radiation-induced pulmonary fibrosis through Nrf2-dependent downregulation of the TGF-β/Smad3 pathways (Bian et al. 2018). These studies suggest that thalidomide exerts its protective effect against pulmonary fibrosis by suppressing bleomycin-induced oxidative stress and the resulting damage to the membrane, thereby preventing inflammation.

#### Current status of preclinical and clinical studies

Over the past few years, there has been a lot of research on repurposing thalidomide in various disorders. However, its use is currently restricted to multiple myeloma and erythema nodosum leprosum. Table 1 represents 40 different studies with highly specific markers studied in IPF models. Despite abundant preclinical data on thalidomide acting against IPF, none have ventured into a clinical trial. Suboptimal confidence in thalidomide in a clinical setting could be attributed to teratogenic effects (Khalil et al. 2020). The multiple mechanisms of action of thalidomide in lung fibrosis urged us to produce this comprehensive report. Anti-inflammatory, immunomodulatory, and anti-angiogenic actions of thalidomide have been shown to be benefit management of aberrant inflammatory responses in IPF. The TNF-α-inhibitory and antifibrotic activity of thalidomide could help mitigate the harmful effects from the abnormal wound healing process during IPF. TGF-β inhibition halts the fibrosis process or progression of the disease (Ye and Hu 2021). See Table 1 for multiple mechanisms of action reported in preclinical and clinical studies, supporting the preventive effect of thalidomide in lung fibrosis.

**Table 1:** Preclinical and clinical trials performed to assess thalidomide / its analogues and their mechanism of action (data are in chronological order)

Sl. no	Description of study/Mechanism of action	Author/References
1	Thalidomide reduced inflammatory markers such as TNF-α, IL-12p40, IL-18, and IL-8 in bronchoalveolar lavage fluid (BALF) of patients with IPF	(Ye 2006)
2	Thalidomide reduced the expression of IL-6, TGF-β1, VEGF, Ang-1 Ang-2, and COL1A1 mRNA in bleomycin-induced mouse model of pulmonary fibrosis	(Tabata et al. 2007)
3	Thalidomide was used to treat cough in IPF patients. Discontinuation of thalidomide resulted in a return of cough within two weeks. Thalidomide is still used as a last resort, when other drugs fail	(Horton et al. 2008)
4	Thalidomide reported to be beneficial in treating IPF in human foetal lung fibroblasts and bleomycin-induced mouse model of pulmonary fibrosis. The mechanisms of action implied were TGF-β pathway and downregulation of α-SMA	(Zhao et al. 2009)
5	Thalidomide reportedly inhibited nitric oxide (NO) in mice model of pulmonary fibrosis	(Nematbakhsh et al. 2009)
6	Thalidomide ameliorated the symptoms of bleomycin-induced mouse model of pulmonary fibrosis through TGF-β and ERK1/2 pathways	(Choe et al. 2010)
7	Thalidomide inhibited experimental peritoneal fibrosis in mice by ameliorating sub-mesothelial thickening and angiogenesis, reducing PCNA- and VEGF-expressing cells, myofibroblasts, and TGF-β-positive cells	(Arai et al. 2011)
8	Thalidomide was reported to reduce inflammatory mediators in BALF in IPF patients in China. This report is a first-of-its-kind in human IPF	(Zhang and Yang 2012)
9	Cough and QoL were significantly improved, although adverse effects such as discomfort, dizziness and constipation were reported in a randomized trial involving 98 patients treated with thalidomide	(Horton et al. 2012)
10	Thalidomide had anti-inflammatory effect in paraquat-induced mouse lung inflammation. It showed reduction of IL-1β, IL-6, TGF)-β1, myeloperoxidase (MPO), NO, and hydroxyproline levels	(Amirshahrokhi 2013)
11	Thalidomide suppressed the inflammatory pathway in the lungs and reduced the release of cytokines and interferons in H1N1-infected BALB/c mice	(Zhu et al. 2014)
12	Thalidomide inhibited bleomycin-induced pulmonary fibrosis in rats by downregulating p-JNK and α-SMA	(Liu et al. 2014)
13	Five thalidomide dithioate analogues showed anti-inflammatory activity by inhibiting TNF-α, NF-κB, and showed free radical scavenging activity by oxygen radical antioxidant capacity (ORAC) and NO inhibition assay	(Talaat et al. 2015)
14	Thalidomide attenuated cisplatin-induced nephrotoxicity, possibly by inhibition of inflammatory reactions (TNF-α, IL-1β, IL-6, TGF-β1, lipid peroxidation, MPO and NO) in the experimental model	(Amirshahrokhi and Khalili 2015)
15	Thalidomide decreased the levels of TNF-α, TGF-β, COL1A1, IL-6 in lung fibrosis by paraquat in Wistar rats	(Li et al. 2015)
16	Thalidomide inhibited inflammation by inhibiting cytokines (TNF-α, IL-1β, IL-6, IL-10 and IL-17) and chemokines such as Keratinocyte chemoattractant (KC), monokine induced by interferon-γ (MIG), and basic fibroblast growth factor (bFGF) in unilateral ureter obstruction	(Bersani-Amado et al. 2016)
17	Thalidomide showed anti-lung fibrosis effect in rat models chiefly by anti-inflammatory and antioxidant effects via regulation of thioredoxin reductase	(Dong et al. 2017a)
18	Thalidomide acted through iNOS and nitric oxide pathways when tested for antinociceptive effects	(Khan et al. 2017)
19	Thalidomide was shown to inhibit lung fibrosis both *in vivo* and in cell lines, chiefly by anti-inflammatory and antioxidant mechanisms	(Dong et al. 2017b)
20	Anti-fibrotic action of thalidomide was through TGF-β1 mechanism and preventing EMT in alveolar epithelial cells through both, Smad-dependent and Smad-independent pathways	(Zhou et al. 2017)
21	Thalidomide analogues exerted anticancer activity in human lung cancer A549 cells through inhibiting VEGF and MMP-3	(El-Aarag et al. 2017)
22	Thalidomide improved psoriasis-like lesions and inhibited the expression of cutaneous VEGF in the imiquimod-induced psoriatic mouse model (BALB/c mice)	(Liu et al. 2018)
23	Thalidomide suppressed renal fibrotic proteins, (E.g.TGF-β1, TβRII, TβRI, smad3, collagen IV, fibronectin) and stimulated the phosphorylation of AMPKα in rats with diabetic nephropathy	(Zhang et al. 2018)
24	Thalidomide decreased ROS levels by inhibition of TGF-β/Smad3 pathway in radiation-induced lung fibrosis in mice	(Bian et al. 2018)
25	Thalidomide attenuated the development of morphine dependence by inhibition of PI3K/Akt and NO signalling pathways in NMRI mice and human glioblastoma T98G cells	(Khan et al. 2018)
26	Pomalidomide was tested for safety and efficacy (phase-2 trial) in interstitial lung disease due to systemic sclerosis,However, pomalidomide did not show statistical significance	(Hsu et al. 2018)
27	Thalidomide down-regulated COX-2 expression in epithelial cells and collagen synthesis, and reduced cough by acting on sensory nerves of the respiratory tract. The test was done in bleomycin-induced lung fibrosis in C57BL/6 mice model	(Rezaie et al. 2019)
28	Thalidomide decreased IFN-γ, IGF-1, IL-6, IL-17, TNF-α, while increasing the levels of IL-10 and TGF-γ. It down-regulated the expression of col1a2, col3a2, MMP-3, MMP-9, MMP-1, TGF-γ, α-SMA, but upregulated the expression of TIMP-1 and Vimentin in TNBS-induced mice colitis	(Chen et al. 2019a)
29	Thalidomide inhibited NO, TNF-α and IL-6 production, reducing portal pressure and increasing the expression of suppressor of cytokine signalling 1 (SOCS1) to prevent cirrhotic cardiomyopathy in rats	(Hosseini-Chegeni et al. 2019)
30	Thalidomide prevented bleomycin-induced systemic sclerosis in a mouse model, acting through TGF-β1/Smad3 pathway. Systemic sclerosis is a type of autoimmune skin fibrosis	(Lu et al. 2020)
31	Thalidomide inhibited the gene promoter activation of CTGF induced by TGF-β1 in human embryonic lung fibroblasts (HELF)	(Wu et al. 2020)
32	Low-dose thalidomide reduced the hyperinflammatory state and calmed down a 45 year-old woman without adverse effects, when given as an adjuvant, with a short-term glucocorticoid, to treat severe lung injury due to COVID-19	(Chen et al. 2020)
33	Thalidomide upregulated T-cell transcriptor (T-bet) in natural killer (NK) cells and down-regulated glycogen synthase kinase-3β (GSK-3β) expression to inhibit lung metastasis of cancer cells	(Miyazato et al. 2020)
34	Thalidomide analogue 6-{4-[(3-(1,3-dioxoisoindolin-2-yl)-2,6-dioxopiperidin-1-yl)methyl]-1H-1,2,3-triazol-1-yl}hexanoic acid (10e) reversed the migratory ability of TGF-β1-induced myofibroblasts, dedifferentiated myofibroblasts to fibroblasts due to cytoskeleton remodelling, and restrained myofibroblast activation by targeting Orai1 and TGF-β1/Smad2/3 signalling pathways	(Tang et al. 2021)
35	Thalidomide, but not lenalidomide or pomalidomide, upregulated spinal microglial IL-10 / β-endorphin expression, to alleviate neuropathic pain	(Deng et al. 2021)
36	Thalidomide suppressed VEGF and Ang-2-induced cell migration and capillary-like tube formation in human umbilical vein endothelial cells (HUVECs). This suggests that thalidomide inhibits angiogenesis via downregulation of VEGF and angiopoietin-2 in Crohn's Disease	(Wang et al. 2021)
37	Thalidomide protected against paraquat-induced lung injury through a miRNA-141/HDAC6/IκBα-NF-κB axis in rat lung fibrosis model and in the RLE-6TN cell model	(Zheng et al. 2021)
38	Thalidomide modulated ER-stress, through TLR4- NF-κB pathway in mouse model of silicosis. Silicosis is a common form of pulmonary fibrosis in China	(Li et al. 2022b)
39	Thalidomide derivative CC-885 was found to be a Basonuclin 2 (BNC2) inhibitor. The transcript BCN2 is responsible for simulating fibrosis signalling in tissues	(Bobowski-Gerard et al. 2022)
40	Thalidomide effectively lowered skin lesions, inflammation and fibrosis related factors in rosacea mice model	(Kang et al. 2022)

Currently approved drugs for IPF are pirfenidone and nintedanib, with similar pros and cons. Pirfenidone is not preferred in case of comorbidities such as renal end-stage disease, because 80% of its clearance is via renal route. Similarly, nintedanib is not recommended for moderate to severe hepatic impairment because most of its metabolism and excretion occur within the hepatic system (Morrow et al. 2022). However, the preference for selection between pirfenidone and nintedanib is based on the patient’s preference on dosing, the adverse drug reactions (ADRs), cost, and insurance coverage (Morrow et al. 2022). Pirfenidone produces ADRs such as nausea, rash, abdominal pain, upper respiratory infection, diarrhoea, fatigue, headache, dyspepsia, dizziness, vomiting, anorexia, gastroesophageal reflux disease (GERD), sinusitis, insomnia, weight loss, and arthralgia (Galli et al. 2017). A combination of pirfenidone with thalidomide has the advantage of mitigating these ADRs. Along with anti-emetic activity, thalidomide has a sedative effect and improves sleep patterns in patients with lung fibrosis (Horton et al. 2012). A recent case study of an IPF patient treated with thalidomide for cough suppression showed an improvement in the patient condition, especially in tackling cough (Haraf et al. 2018). However, the efficacy of relieving or reversing fibrosis has not been tested in the case. Although thalidomide has been used for treating cough in IPF, it is only used when other drugs fail. The recently published case study is one such example (Haraf et al. 2018). Previously, a randomized study included only 24 patients with IPF and cough (Horton et al. 2012), and another study had only 11 patients having cough in IPF (Horton et al. 2008). The recent meta-analysis on thalidomide did not include data from patients with IPF (Xie et al. 2022). Thus, thalidomide has not yet undergone thorough clinical investigations owing to its teratogenic history. The preclinical effect of thalidomide is mainly mediated by inhibition of TFG-β, TNF-α, ILs and by antioxidant mechanisms. Significant advancement in diagnosis and prognosis of IPF, and availability of improved biomarkers for statistical comparison of therapeutic output, make clinical trials appropriate and timely (Chen et al. 2019b; Christe et al. 2019; Hochhegger et al. 2019; Tomassetti et al. 2020). Hence, based on the above highlights, we propose a prospective clinical investigation for thalidomide in the treatment of IPF.

## Conclusion

There has been significant progress in thalidomide research, paving the way for its potential repurposing in inflammatory and neoplastic conditions. Thalidomide, which had once been revoked of its title of wonder drug, has managed to find a place in healthcare for the treatment of multiple myeloma and erythema nodosum leprosum. Despite the uncertainty regarding its potential in pulmonary fibrosis, a growing body of evidence substantiates the rationale of repurposing thalidomide for treating and slowing down the onset of fibrosis. The discovery of the thalidomide-binding protein, cereblon, was a breakthrough study that helped understand, to a certain extent, the mechanism of action of thalidomide at the molecular level. With recent information on the role of cereblon in the progression of IPF and newer biomarkers established for clinical trial assessments, thalidomide will have a promising role in preventing fibrosis, warranting future investigations.

## Data Availability

As this is a review manuscript, there is no data generated.
